# Low dose IV buprenorphine inductions for patients with opioid use disorder and concurrent pain: a retrospective case series

**DOI:** 10.1186/s13722-023-00392-z

**Published:** 2023-06-01

**Authors:** John P. Murray, Geoffrey Pucci, George Weyer, Mim Ari, Sarah Dickson, Angela Kerins

**Affiliations:** 1 Department of Medicine, University of Chicago, 5841 S Maryland Ave, Chicago, IL 60637 USA; 2 Department of Pharmacology, University of Chicago, 5841 S Maryland Ave, Chicago, IL 60637 USA

**Keywords:** Opioid use disorder, Low dose induction, Opioid agonist therapy, Microinduction, Addiction medicine, Buprenorphine

## Abstract

**Background:**

Hospitalizations are a vital opportunity for the initiation of life-saving opioid agonist therapy (OAT) for patients with opioid use disorder. A novel approach to OAT initiation is the use of IV buprenorphine for low dose induction, which allows patients to immediately start buprenorphine at any point in a hospitalization without stopping full agonist opioids or experiencing significant withdrawal.

**Methods:**

This is a retrospective case series of 33 patients with opioid use disorder concurrently treated with full agonist opioids for pain who voluntarily underwent low dose induction at a tertiary academic medical center. Low dose induction is the process of initiating very low doses of buprenorphine at fixed intervals with gradual dose increases in patients who recently received or are simultaneously treated with full opioid agonists. Our study reports one primary outcome: successful completion of the low dose induction (i.e. transitioned from low dose IV buprenorphine to sublingual buprenorphine-naloxone) and three secondary outcomes: discharge from the hospital with buprenorphine-naloxone prescription, self-reported pain scores, and nursing-assessed clinical opiate withdrawal scale (COWS) scores over a 6-day period, using descriptive statistics. COWS and pain scores were obtained from day 0 (prior to starting the low dose induction) to day 5 to assess the effect on withdrawal symptoms and pain control.

**Results:**

Thirty patients completed the low dose induction (30/33, 90.9%). Thirty patients (30/33, 90.9%) were discharged with a buprenorphine prescription. Pain and COWS scores remained stable over the course of the study period. Mean COWS scores for all patients were 2.6 (SD 2.8) on day 0 and 1.6 (SD 2.6) on day 5. Mean pain scores for all patients were 4.4 (SD 2.1) on day 0 and 3.5 on day 5 (SD 2.1).

**Conclusions:**

This study found that an IV buprenorphine low dose induction protocol was well-tolerated by a group of 33 hospitalized patients with opioid use disorder with co-occurring pain requiring full agonist opioid therapy. COWS and pain scores improved for the majority of patients. This is the first case series to report mean daily COWS and pain scores over an extended period throughout a low dose induction process.

## Background

In 2021 there were an estimated 107,622 deaths in the United States from drug overdoses, representing a 15% increase from the year prior and a 30% increase from 2019 [1]. Opioid overdoses constitute the vast majority of all drug overdoses, a sprawling, uncontrolled public health crisis that has effectively worsened year over year for two decades and has contributed to an overall decline in life expectancy in the United States [2, 3]. Studies show that inpatient hospitalizations are a vital moment in initiating treatment and linking patients with opioid use disorder (OUD) to outpatient care [4–11]. Opioid agonist therapy (OAT: buprenorphine or methadone) has been associated with a profound mortality benefit for patients with OUD, arguably greater than any other class of medication [12–15]. Unfortunately, both are under-prescribed and, in many cases, not in the pharmacological armamentarium of inpatient providers or hospital systems [16–20].

There are many reasons for these missed opportunities including stigma, provider hesitancy or inexperience, and federal regulations limiting methadone prescribing for OUD in the outpatient setting [21–26]. An additional barrier to buprenorphine treatment in the hospital setting is that, conventionally, patients with OUD need to be in mild-moderate withdrawal or fully abstinent from all opioids for a period of time prior to induction: the process of transitioning from a full-agonist opioid to the partial agonist buprenorphine [27]. Traditionally, all short-acting opioids are held for 12–24 h prior to induction, while long-acting opioids such as methadone or non-pharmaceutical fentanyl require a washout period of two-five days. This is done to avoid precipitated opioid withdrawal (POW). POW has been shown to decrease both provider and patient willingness to administer or accept buprenorphine in the future [28, 29]. Buprenorphine induction is especially challenging in the hospital setting where 65% of patients report experiencing pain within the past 24 h [30, 31] and 51% of nonsurgical patients are treated with opioids at some point during their hospitalization [32].

Low dose induction is the practice of administering very low doses of buprenorphine at fixed intervals with incremental dose increases such that the displacement of full agonist opioids at the µ-opioid receptor is so gradual that POW symptoms do not occur or are clinically attenuated [33, 34]. It is increasingly utilized and studied in the inpatient and ED settings as detailed in a number of case reports and case series, including a recent 68-person retrospective cohort study using sublingual buprenorphine and a 59-person retrospective study also describing an IV protocol [35–47]. Hospitalized patients, many of whom suffer from pain that necessitates the regular administration of full-agonist opioids, are a uniquely challenging population for buprenorphine induction. Low dose induction is particularly useful in the inpatient setting, where the need for pain management using full agonist opioid medications is frequently a barrier to successful induction.

In the United States, buprenorphine is orally available in sublingual film and tablet forms, with the most common low dose being 2 mg. As shown in Table 1, normal initial low dose induction doses start at 0.2 to 0.5 mg of sublingual buprenorphine, which requires cutting 2 mg strips into smaller pieces. Because altering medications was prohibited by our hospital system, we turned to IV buprenorphine which comes in doses that correspond to the appropriate milligram range for low dose induction. The bioavailability of sublingual buprenorphine ranges from 30 to 50% and has similar pharmacokinetics to IV buprenorphine [48, 49]. There was one previously published case report that described the use of IV buprenorphine for low dose induction that helped shape the basis for our work [38]. Traditionally, IV buprenorphine has been used and studied in the hospital setting for acute pain control, not OAT. Our strategy relied upon using IV buprenorphine for 2–4 days at the start of the low dose induction period and then transitioning to sublingual buprenorphine-naloxone (usually Suboxone) to complete the process.

**Table 1:** Summary of published buprenorphine low dose induction protocols

	Total daily buprenorphine dosage (mg)	Day 1	Day 2	Day 3	Day 4	Day 5
Bernes method		0.2 SL	0.2 SL	2.8 SL	4.5 SL	5 SL
Hammig (2016)		0.2 SL	0.8 SL	1.2 SL	1.8 SL	2.0 SL
Terasaki (2019)		0.5 SL	1.0 SL	2.0 SL	4.0 SL	8.0 SL
Crane (2020)		0.1 IV	1.1 IV	1.2 IV	1.6 IV	1.5 IV
Teck (2021)		0.4 SL	0.4 SL	0.8 SL	1.2 SL	1.6 SL
Jablonski (2022)		1.0 IV	1.8 IV	4.0 SL	–	–

In this paper we present a retrospective case series examining 33 hospitalized patients with OUD and concurrent pain requiring full agonist opioids who underwent low dose induction utilizing IV buprenorphine. Data was abstracted over a 10-month period from May 1st, 2021, to March 1st, 2022, at an urban, tertiary academic medical center with a Level I Trauma Center. Additionally, we report average daily COWS and pain scores throughout the low dose induction process, a first for a case series of this size.

## Methods

We performed a retrospective chart review of patients who received IV buprenorphine from May 1st, 2021, to March 1st, 2022. Inclusion criteria were use of IV buprenorphine for the purposes of low dose induction and receiving full agonist opioids for pain control during the day prior to induction. Exclusion criteria were use of IV buprenorphine for any other reason and not requiring full agonist opioids for pain control the day prior to induction. We also excluded patients who were receiving methadone only for OUD and did not have co-occurring pain. When appropriate patients were identified, researchers performed a manual chart review to obtain all clinical opiate withdrawal scores (COWS) and pain scores over a 6-day period, starting at midnight the day prior to the initial IV buprenorphine administration and extending for the first 5 days of the low dose induction. COWS measures opioid withdrawal severity on an ascending scale from 0 to 48, while pain scores were self-reported on a scale of 0–10 [50]. Both were obtained by nursing staff and entered into electronic health record flowsheets.

Review of patient charts identified 2 distinct low dose induction strategies: a “standard” and a “slow” regimen (Table 2). The standard regimen (27 patients) entailed induction with 0.15 mg IV buprenorphine every 6 h on day 1 with dose titration to 0.3 mg and then 0.4 mg on subsequent days. The slow regimen (6 patients) was characterized by starting with 0.15 mg IV buprenorphine but dosed every 12 h, often in the setting of transitioning from methadone or high-dose full agonist opioids. Patients in both groups were transitioned from IV buprenorphine to sublingual buprenorphine-naloxone 2/0.5 mg every 12 h on day 3 or 4 based on the clinical judgment of the provider. Full agonist opioids were continued throughout and beyond the microinduction process as clinically appropriate. Choice of low dose induction strategy was provider-dependent based on the degree of recent opioid use and the need for concurrent pain management.

**Table 2:** Low dose induction regimens

	Definition
Standard regimen	Intravenous (IV) buprenorphine was scheduled every 6 h in 24-h period with the goal of transitioning to sublingual (SL) buprenorphine by day 3 or 4 of therapy Day 1: IV buprenorphine 0.15 mg every 6 h (~ 1–2 mg SL dose) Day 2: IV buprenorphine 0.3 mg every 6 h (~ 3–4 mg SL dose) Transition to SL on day 3 or 4: Day 3: IV buprenorphine 0.4 mg every 6 h (~ 3.5–5 mg SL dose) or SL buprenorphine/naloxone 2/0.5 mg BID then 4 mg TID on the next day Day 4: IV buprenorphine 0.5 mg every 6 h (~ 4–6 mg SL dose) or SL buprenorphine/naloxone 2/0.5 mg BID then 4 mg TID on the next day
Slow regimen	IV buprenorphine was scheduled every 12 h in a 24-h period with the goal of transitioning to SL buprenorphine by day 3 or 4 of therapy Day 1: IV buprenorphine 0.15 mg every 12 h (0.5–1 mg SL dose) Day 2: IV buprenorphine 0.3 mg every 12 h (~ 1–2 mg SL dose) Transition to SL on day 3 or 4: Day 3: IV buprenorphine 0.4 mg every 12 h (~ 1.75–2.5 mg SL dose) or SL buprenorphine/naloxone 2/0.5 mg BID then 4 mg TID on the next day Day 4: IV buprenorphine 0.5 mg every 12 h (~ 2–3 mg SL dose) or SL buprenorphine/naloxone 2/0.5 mg BID then 4 mg TID on the next day

Two separate researchers performed a complete chart review and compared data to ensure accuracy. Daily COWS and pain scores were aggregated in 24-h intervals using mean values to 1 decimal, given the number of data points and high variance, particularly with regards to pain scores. Day 0 represents the time from midnight the day prior until buprenorphine administration on day 1 to distinguish pre and post buprenorphine COWS and pain scores. Subsequent days were delineated in midnight-to-midnight intervals, which means day 1 scores do not represent a full 24-h period.

All charts were reviewed to determine if patients successfully completed the microinduction process and, if not, the primary reason for discontinuation. Because this is a relatively small single-center retrospective study, descriptive statistics are used to analyze the data. This project received a formal Determination of Quality Improvement status according to University of Chicago Medicine’s institutional policy. As such, this initiative was deemed not human subjects research and was therefore not reviewed by the Institutional Review Board. This paper follows the CARE reporting standards for case series reports.

## Results

During the study period, 60 patients were administered IV buprenorphine, of which 49 met low dose induction criteria and 33 were treated with full opioid agonists for pain in the 24 h prior to induction. Table 3 includes patient demographics, collated results of initial urine toxicology testing, opioid requirement in morphine milligram equivalents (MME) for the 24 h prior to the start of induction, average hospital day when induction was started, number of patients who completed the induction, and the number of patients discharged with a buprenorphine prescription. Most patients were male (20/33, 60.6%) and African American (24/33, 72.7%). The majority of the 33 patients were admitted to either the trauma service (12 patients) or a general medicine team (10 patients). The median MME dose in the 24 h prior to induction was 30.8 for all patients.

**Table 3:** Patient Characteristics

	Total population (n = 33)
Age, years (median, IQR)	58 (41–64)
Male, [n (%)]	20 (60.6)
Race, [n (%)]	
Black/African American	24 (72.7)
White	9 (27.3)
QTc ≥ 500 ms, [n (%)]	4 (12.1)
UDS on admit, [n (%)]	22 (66.7)
Opiates	22 (66.7)
Benzodiazepines	6 (18.2)
Marijuana	1 (3.0)
Cocaine	8 (24.2)
Barbiturates	1 (3.0)
Amphetamines	2 (6.1)
Methadone	6 (18.2)
Oxycodone	3 (9.1)
Required opioids 24 h prior to microinduction, [n (%)]	33 (100.0)
Median MME (median, IQR)	30.8 (15–60)
Admission day IV buprenorphine was started on [median (IQR)]	4 (2–7)
Time from last IV to first SL dose, hours [median (IQR)]	10.3 (7.8–12.5)
Day of microinduction transitioned to SL (median, IQR)	4 (3–4)
Methadone cross taper with buprenorphine, [n (%)]	7 (21.2)
Last day of methadone dose (median, IQR)	3 (2–4)
Required dose adjustment, [n (%)]	8 (24.2)
Completed buprenorphine induction therapy, [n (%)]	30 (90.9)
Standard regimen, [n (%)]	25 (92.6)
Slow regimen, [n (%)]	5 (83.3)
Discharge with buprenorphine prescription, [n (%)]	30 (90.9)
Buprenorphine only, [n (%)]	19 (57.6)
Buprenorphine with scheduled opioids, [n (%)]	3 (9.1)
Buprenorphine with as needed opioids, [n (%)]	8 (24.2)

IQR: Inter Quartile Range; MS: Milliseconds; UDS: Urine Drug Screen; MME: Morphine Milligram Equivalents; IV: Intravenous; SL: Sublingual; PO: by mouth

The low dose induction was successful for the majority of patients, with 90.9% of all patients completing the 3–5-day process (Table 3). Rates were similar between the two groups with 92.6% of patients completing the standard regimen and 83.3% completing the slow regimen. There were three instances of treatment failure: 1 self-directed discharge (slow regimen), 1 patient resumed their prior to admission methadone due to improvement of a previously-prolonged QTc (slow regimen), and 1 patient stopped due to worsening of acute on chronic cancer pain (standard regimen). Thirty patients (90.9%) were discharged from the hospital with a buprenorphine prescription. Of these, eleven (11/30, 36.7%) received a prescription for either as needed (8/30, 26.7%) or scheduled (3/30, 10%) full opioid agonists.

Additionally, we identified all patients who required an adjustment to their initially ordered low dose induction regimens. There were 10 total patients (30.3%) who required a change to the initially ordered protocol: 10 (37.0%) in the standard regimen and 1 (16.7%) in the slow regimen. We identified 5 primary reasons for dosage adjustment (Table 4): worsening withdrawal symptoms (2 patients), uncontrolled pain (3 patients), patient safety reasons including somnolence or inability to tolerate sublingual medications (2 patients), self-directed discharge (1 patient), and incorrectly placed orders (2 patients). One patient discontinued the low dose induction due to concerns about both pain and withdrawal.

**Table 4:** Reasons for dose adjustment from initial regimen

	Standard regimen (n = 27)	Slow regimen (n = 6)
Opioid withdrawal induced, n	1	1
Uncontrolled pain, n	3	0
Patient safety (somnolence, inability to swallow, intubation), n	2	0
Self-directed discharge, n	1	0
Medication ordering error, n	2	0

During the low dose induction, COWS and pain scores downtrended between day 0 and day 5 for most patients and were on-average low throughout the process (Table 5). Mean COWS scores were 2.6 (SD 2.8) on day 0 and 1.6 (SD 2.6) on day 5 for all patients. Mean pain scores were 4.4 (SD 2.1) on day 0 and 3.5 (SD 2.1) on day 5 for all patients. On average, the low dose induction was started on day 4 of hospitalization.

**Table 5:** Documented COWS and pain scores

	COWS score			Pain score		
	Standard regimen (n = 27)	Slow regimen (n = 6)	Total (n = 33)	Standard regimen (n = 27)	Slow regimen (n = 6)	Total (n = 33)
Day 0, mean (SD)	2.8 (3.2) N = 13	2.1 (2.0) N = 6	2.6 (2.8) N = 19	4.5 (2.1) N = 26	3.8 (1.9) N = 6	4.4 (2.1) N = 32
Day 1, mean (SD)	2.3 (2.4) N = 17	3.5 (3.6) N = 4	2.6 (2.6) N = 21	4.3 (2.7) N = 26	3.9 (2.3) N = 6	4.2 (2.6) N = 32
Day 2, mean (SD)	2.0 (2.4) N = 15	0.7 (0.6) N = 4	1.7 (2.2) N = 19	4.2 (2.0) N = 26	3.2 (2.0) N = 6	4.0 (2.0) N = 32
Day 3, mean (SD)	1.6 (1.4) N = 13	0.9 (0.9) N = 5	1.4 (1.3) N = 18	3.5 (2.6) N = 26	6.0 (1.9) N = 6	4.0 (2.6) N = 32
Day 4, mean (SD)	1.0 (1.4) N = 13	0.9 (0.9) N = 4	0.9 (1.3) N = 17	3.5 (2.3) N = 24	3.5 (2.0) N = 5	3.5 (2.2) N = 29
Day 5, mean (SD)	1.8 (2.9) N = 11	2.7 (1.7) N = 3	1.6 (2.6) N = 14	3.6 (2.2) N = 19	2.9 (1.7) N = 4	3.5 (2.1) N = 23

SD: standard deviation

## Discussion

This retrospective case series details the results of a low dose IV buprenorphine induction protocol at a tertiary, academic medical center. Thirty-three patients with OUD requiring full agonist opioids for pain control underwent low dose induction during a 10-month period. Researchers retrospectively recorded daily COWS and pain scores for patients during the low dose induction process, which are reported in daily averages, a first for a case series of this size. There were two low dose induction strategies identified: a standard and a slow regimen. These strategies were not prefabricated but rather evolved experientially to provide customized care for patients who were taking a spectrum of full agonist opioids at a wide range of doses and half-lives. Notably, the low dose IV induction protocol described by Jablonski et al. was published after the completion of this case series and did not inform these design protocols [41]. Of the 33 patients studied, 30 (90.9%) successfully completed the process, with only 1 self-directed discharge during the study period, which is striking given high rates of for this population [51, 52]. Additionally, 90.9% of all patients were discharged with a buprenorphine prescription.

Recorded COWS and pain scores remained stable or decreased throughout the low dose induction period for the majority of patients. The mean COWS score on day 0 was 2.6 (SD 2.8) and by day 5 was 1.6 (SD 2.6), while the mean pain score on day 0 was 4.4 (SD 2.1) and by day 5 was 3.5 (SD 2.1), suggesting that the low dose induction process did not cause worsening withdrawal or complicate pain control, even for a cohort of patient with active pain control needs. By reporting daily COWS and pain scores throughout the induction period, this study hopes to demonstrate that low dose induction is generally well-tolerated, even for patients requiring full agonist opioids for pain, opening the door to buprenorphine treatment for a range of hospitalized patients who might not otherwise be considered candidates due to ongoing full agonist opioid requirements or due to recent use of methadone or non-pharmaceutical fentanyl.

This paper has a number of limitations including imprecision in the reporting of data: day 1 begins with the first administration of IV buprenorphine but day 2 resets at a midnight-to-midnight interval. This means that day 0 encompasses a greater than 24-h period and day 1 a shorter and variable time period. Another limitation is lack of information about adjunctive medications such as ondansetron, dicyclomine, clonidine, and loperamide, which are frequently used to mitigate opioid withdrawal symptoms.

Additionally, there was variability in the number and frequency of pain and COWS scores recorded (Table 5), with less data available later in the low dose induction, making results vulnerable to skew. However, we believe this would bias our results towards over-reporting pain severity in the later days of the low dose induction as nurses would typically perform fewer bedside assessments for patients demonstrating improved symptoms, leaving more data to be collected from patients with ongoing withdrawal or pain. Lastly, the regimens themselves were not perfectly standardized, with small variations in buprenorphine dosing and frequency, making them susceptible to ordering error (2 patients).

As the opioid epidemic worsens, hospitalizations are increasingly understood as a crucial opportunity to engage patients with opioid use disorder and initiate OAT. By and large, hospital systems fall short of providing evidence-based care to patients with OUD, with prior studies suggesting that 11–15% of patients receive OAT during hospitalizations [6, 53]. The growing awareness of this shortcoming has led to a profusion of both specialized addiction medicine consult services and general internist-led efforts to manage OUD in the hospital setting [9, 11, 54–56]. However, challenges for the safe initiation of OAT remain, including the need for patients to be in mild-moderate withdrawal prior to induction, pressure to discharge patients for length-of-stay reasons, and the proliferation of non-pharmaceutical fentanyl, which may make standard inductions more challenging [57, 58].

## Conclusions

Low dose induction enables patients with OUD to start buprenorphine immediately upon hospital admission, without waiting for withdrawal symptoms to develop, and to receive full agonist opioids at any point during or after the induction process. Previously restricted to the realm of short case reports, low dose induction is increasingly coming into the clinical mainstream, and we hope it will soon be in the pharmacological toolkit of most hospital systems. The use of IV buprenorphine at the onset of low dose induction is easily protocolized, affordable, and pharmacologically precise [59]. It averts the need to cut buprenorphine-naloxone strips, attenuates precipitated withdrawal from long-acting opioids, and allows for the continuous use of full agonists opioids for pain management. As we look for new solutions to the evolving opioid epidemic, we believe IV buprenorphine will be an essential tool for the management of OUD in patients with concurrent pain in the hospital setting.

## Data Availability

All supplemental data and materials are available for review upon request.

## Abbreviations

OAT: Opioid agonist therapy
COWS: Clinical opiate withdrawal scale
OUD: Opioid use disorder
MOR: µ-Opioid receptor
POW: Precipitated opioid withdrawal
